# Supplementary material for: Collaborative intelligence in AI: Evaluating the performance of a council of AIs on the USMLE
Source: PLOS Digit Health. 2025 Oct 9;4(10):e0000787. doi: 10.1371/journal.pdig.0000787 (PMC12510544; doi:10.1371/journal.pdig.0000787)
Supplement: S2 File — (ZIP) [file pdig.0000787.s002.zip › S2 Data Raw data files containing unprocessed question inputs and LLM outputs including transcripts of deliberations/STEP3_Question-76-1_Type-MC-NJ.docx]

**Facilitator** **[Round 1]:** A 41-year-old woman is brought to the emergency department by her husband because of nausea and dizziness that began suddenly 4 hours ago. She says she feels like the room is spinning. She has not vomited. She recalls receiving a blow to the left side of her neck 1 week ago when her dog jumped on her, but she has had no ill effects from that incident. Medical history is unremarkable. She takes no medications. Vital signs are normal. Physical examination shows mild ptosis of the left eyelid. The right pupil measures 4 mm in diameter and the left pupil measures 2 mm in diameter. Both pupils are reactive to light. Ocular motility is full. Nystagmus is present in both eyes on left lateral gaze. Left corneal reflex is diminished. Sensation to pinprick and cold is reduced over the left side of the face, right side of the chest, abdomen, and right upper and lower extremities. Which of the following pathophysiologic processes most accurately explains this patient's left-sided ptosis and decreased pupil size? (A) Parasympathetic overactivity (B) Parasympathetic underactivity (C) Postsynaptic neuromuscular junction defect (D) Presynaptic neuromuscular junction defect (E) Sympathetic overactivity (F) Sympathetic underactivity

**AI_D:** (1) RESTATING THE QUESTION: We have a case of a 41-year-old woman presenting to the emergency department with symptoms of nausea, dizziness, and vertigo. She also mentioned a history of trauma to her neck recently. She now shows signs of unilateral ptosis and miosis, along with diminished sensation to pinprick and cold on the left side of her face and contralateral trunk and limbs. The question is asking which pathophysiologic process is causing her left-sided drooping of the eyelid (ptosis) and reduced pupil size (miosis). (2) PLANNING: The list of potential explanations provided includes abnormalities in parasympathetic or sympathetic activity, as well as defects at the neuromuscular junction. Specifically, these possibilities represent either overactivity or underactivity of the parasympathetic or sympathetic systems, and either postsynaptic (receptor-side) or presynaptic (nerve terminal-side) defects at the neuromuscular junction. We, therefore, need to first understand the roles these systems play in eye function and sensation. Considering her neck trauma, it may be possible that damage could have occurred to the sympathetic trunk on that side, leading to a disruption in sympathetic input. (3) CLINICAL REASONING: Ptosis (eyelid drooping) and miosis (constriction of the pupil) are classic signs of Horner's syndrome, which is caused by a disruption of sympathetic innervation to the eye. Sympathetic nerves maintain eyelid elevation (preventing ptosis) and promote pupil dilation (countering miosis). Thus, sympathetic underactivity can lead to ptosis and miosis. The history of trauma to her neck could have damaged the sympathetic trunk, producing these signs. The condition is also associated with decreased sweating (anhydrosis) on the affected side of the face, which was not directly observed here. The additional symptoms of reduced sensitivity to pinprick and temperature stimuli suggest a lesion involving the spinothalamic tract, possibly due to its close proximity to the sympathetic fibers that could have been similarly affected by her neck injury. (4) CONCLUDING: Therefore, when connecting all these clues provided by the symptoms and the relevant clinical knowledge, the woman's condition was likely induced by the damage to the sympathetic trunk (secondary to her neck trauma), which led to a decrease in sympathetic activity on that side. (5) RESPONSE: Thus, the pathophysiologic process most likely explaining this patient's symptoms is (F) Sympathetic underactivity.

**AI_E:** RESTATING THE QUESTION: What is the primary pathological process that could explain why a woman who received a blow to the left side of her neck a week ago is now presenting with symptoms such as nausea, dizziness, and the feeling of the room spinning, as well as left eyelid ptosis, a diminished left corneal reflex, and decreased left pupil size? There is also a noticeable change in sensory perception on her left face and right torso and limbs. Is this a case of sympathetic or parasympathetic nervous system disruption, or could it be a defect at the neuromuscular junction? PLANNING: To answer this question, we are considering either a neuropathological issue that affects the sympathetic or parasympathetic nervous system, or a pathophysiological process affecting the neuromuscular junction. Inspecting these symptoms, they seem to suggest a Horner's syndrome type of pattern, which involves the sympathetic pathway disruption. Mild ptosis, decreased pupil size or miosis, and diminished corneal reflex are seen in Horner's syndrome. However, we would need to assess the injury she experienced a week ago and its potential impact on such a condition. CLINICAL REASONING: Horner's syndrome is a relatively common condition characterized by miosis, ptosis, and anhydrosis. These symptoms are typically unilateral, which aligns with our patient's physical examination findings. Horner's syndrome occurs due to a disruption in the sympathetic pathways somewhere from the hypothalamus to the eye. The blow she received a week ago on her neck, while it appeared to have no ill effects initially, may have caused a form of trauma to the sympathetic chain in the neck. This hypothesis stands if we link the sensory deficit on the left face (same side as the ptosis and miosis) and on the right side of the body (as it should be in a lateral medullary syndrome due to an interruption of the spinothalamic tract). CONCLUDING: While neurological effects might not have been immediately visible, the combination of symptoms is indicative of a complicated region-specific neurological dysfunction. The most critical issue seems to be sympathetic underactivity resulting from a disruption somewhere along the oculosympathetic pathway. RESPONSE: Therefore, the most accurate explanation of this patient's left-sided ptosis and reduced pupil size, given the information available and considering the circumstances of her trauma, seems to point towards Sympathetic underactivity (F), which may be indicative of Horner's syndrome.

**AI_C:** (1) RESTATING THE QUESTION: The query seeks to understand the pathophysiologic process that best correlates with the observed ptosis (a term used to describe the drooping of an eyelid) and miosis (referencing the contraction of the pupil) on the patient's left side. The options provided include different states of activity (overactivity or underactivity) of the autonomic nervous system branches - sympathetic and parasympathetic - and the possibility of a defect in the neuromuscular junction present at either postsynaptic (after the synaptic cleft) or presynaptic (before the synaptic cleft) level. (2) PLANNING: To identify the cause, we shall primarily consider how the mechanisms under the listed causes could contribute to the symptoms mentioned. The episode of a blow to the neck might have resulted in a lesion or injury impacting either nervous signaling or muscular function. The autonomous nervous system, which comprises of the sympathetic and parasympathetic branches, governs functions like pupil dilation and eyelid elevation. A disruption in the balance of activation in these branches or an abnormality in the neuromuscular junction which relays signals from the nerve to the muscle, could be responsible for the observed symptoms. (3) CLINICAL REASONING: Starting from our current assumptions, here's how our findings align: Miosis (pupil contraction) on the left and ptosis (drooping of eyelid) are controlled by the parasympathetic and sympathetic systems respectively. The sympathetic system generally causes pupillary dilation and eyelid elevation, while the parasympathetic system prompts pupillary constriction. Therefore, overactivity of the parasympathetic system or underactivity of the sympathetic system could lead to the observed miosis and ptosis. However, the presence of nystagmus, reduced left corneal reflex, and diminished sensation to pinprick and cold stimuli over the left side of the face and the right side of the body additionally suggest a disruption of cortical sensory pathways, pointing towards more of a central lesion. (4) CONCLUDING: Taking all these findings into account, we can narrow down the possible pathophysiological causes. The findings are suggestive of Horner’s syndrome, where the symptoms of ptosis, miosis, and anhidrosis (reduced sweating) occur due to damage to the sympathetic nerve pathways that run from the brain to the face. The symptoms along with sensory changes suggest a lesion of the lateral medullary (Wallenberg) syndrome affecting the PICA (posterior inferior cerebellar artery) vascular territory, which is known to present with these coupled symptoms. (5) RESPONSE: Thus, keeping all findings from the clinical examination and reasoning in view, sympathetic underactivity (F) is the pathophysiologic process that most accurately explains this patient's left-sided ptosis and decreased pupil size.

**AI_B:** 1) RESTATING THE QUESTION: We have a 41-year-old female who presents to the emergency department with sudden onset of nausea and dizziness, particularly a sensation of the room spinning. She also reports having endured some injury to her neck from her dog a week before but without any initial adverse impact. The physical examination reveals a series of abnormal findings, most crucially a smaller left pupil compared to the right, mild left eye droop (ptosis), an impaired left corneal reflex, and reduced sensation to certain stimuli on one half of her face and the opposite half of her body. The query is about the pathophysiology that would precisely explain her left side eye droop and smaller pupil size. 2) PLANNING: One major assumption here is that the dog's jumping and hitting her neck might have caused an injury which led to the neurological signs we are currently observing. Another assumption could be another underlying issue, like a brain lesion or carotid artery dissection. In this scenario, the change in pupillary size and the reduced sensation point to an injury of the cranial nerves. The question debates these neurological changes in terms of parasympathetic and sympathetic over/underactivity, or defects at the neuromuscular junction. 3) CLINICAL REASONING: Let's look into each of these. Parasympathetic overactivity and underactivity could be ruled out because parasympathetic nerve distribution does not account for all the symptoms, including reduced sensation to pinprick over the face, chest, abdomen and extremities. It is not likely a postsynaptic or presynaptic neuromuscular junction defect like myasthenia gravis (C & D) as these conditions typically present with fatigability and more systemic signs like generalized muscle weakness, often involving the eye and bulbar muscles but not reducing sensation. The decreased pupil size (miosis) and the ptosis suggests a problem with the sympathetic innervation to the eye, the "Horner's syndrome" which happens when there is an interruption in the sympathetic nerves that go to the eye. The lesion that this woman has is in a location known as the lateral medulla of the brainstem, also called Wallenberg's syndrome, due to her other symptoms such as the loss of pain and temperature sensation on one side of the face and the opposite side of the body. This indicates a lesion to the spinothalamic tract as it crosses in the spinal cord but before it crosses in the brainstem, which often arises due to ischemic stroke in the posterior cerebellar artery. 4) CONCLUDING: Therefore, the pathogenic process that correlates most accurately with this woman's left-sided ptosis and decreased pupil size is not accounted by parasympathetic anomalies or defects at the neuromuscular junctions but by an issue with the sympathetic innervation to her eye. 5) RESPONSE: In conclusion, this patient's signs and symptoms most accurately suggest (F) Sympathetic underactivity.

**AI_A:** RESTATING THE QUESTION: The question fundamentally asks us to explain the most likely underlying pathophysiology causing ptosis (drooping of the upper eyelid) and smaller pupil size on the left side of this 41-year-old woman. This woman is displaying a cluster of symptoms, notably nausea, dizziness, mild ptosis on the left eye, reduced pupil size on the same side, decreased corneal reflex, and altered sensation on one side of her face and the opposite side of her body. A range of pathophysiologic processes have been given as potential explanations, which involve autonomic nervous system activity and neuromuscular junction defects. PLANNING: To approach this question, it is necessary first to identify that the given symptoms indicate a specific clinical syndrome, such as Horner's syndrome, which is characterized by ipsilateral miosis (pupil constriction), mild ptosis, and loss of sweating (anhydrosis). Given the patient's history, it is reasonable to hypothesize that the trauma to the left side of her neck may have resulted in an injury to the sympathetic trunk, resulting in Horner's syndrome. The given options reflect different aspects of sympathetic and parasympathetic activity, along with neuromuscular junction defects. We should remember that parasympathetic activity generally constricts the pupil (miosis) and controls eyelid elevation through the levator palpebrae superioris muscle, while the sympathetic system dilates the pupil (mydriasis) and controls the superior tarsal muscle contributing to lid elevation. So, an overactive parasympathetic system or an underactive sympathetic system could explain the symptoms. Alternately, a defect of the neuromuscular junction, presynaptic or postsynaptic, might result in muscle weakness causing ptosis, but that would not typically affect pupil size or sensation. CLINICAL REASONING: The combination of symptoms observed in this patient suggests a disruption in the sympathetic innervation on the left side, sometimes referred to as Horner’s syndrome. This is based on the manifestations of miosis (constricted pupil), and ptosis (drooping eyelid), both resulting from impaired ocular sympathetic innervation. Given the reduced sensation on the right side of the body, the lesion might be located in the brainstem, affecting the descending sympathetic tract (a condition also known as lateral medullary syndrome or Wallenberg syndrome). The sympathetic nervous system generally maintains the upper eyelid position, dilates the pupils, and inhibits sweating. Any harm or disruption to these fibers can lead to the set of changes seen in Horner's syndrome. Unlike Horner's syndrome, a neuromuscular junction defect typically wouldn't present with changes in pupil size or sensation. CONCLUDING: Considering the symptoms of miosis (constricted pupil), ipsilateral ptosis (eyelid droop), and decreased sensation contralaterally, the symptoms are suggestive of an interruption of the sympathetic fibers that could have been caused by the injury received a week ago. RESPONSE: Given these clinical signs and interpreting the relationship between the patient's symptoms and ongoing pathophysiologic processes, the most accurate explanation for this patient's symptoms would be (F) Sympathetic underactivity.

**Facilitator** The question is asking for the most accurate pathophysiologic process that explains the left-sided ptosis (drooping of the eyelid) and decreased pupil size in a 41-year-old woman who recently experienced a blow to the left side of her neck. The woman's symptoms, which include nausea, dizziness, mild ptosis of the left eyelid, a smaller left pupil, diminished left corneal reflex, and reduced sensation on the left side of her face and the right side of her body, suggest a disruption in the sympathetic innervation on the left side, often referred to as Horner's syndrome. This syndrome is characterized by miosis (constricted pupil), ptosis (drooping eyelid), and loss of sweating (anhydrosis), all resulting from impaired ocular sympathetic innervation. The reduced sensation on the right side of the body suggests a lesion in the brainstem, affecting the descending sympathetic tract, a condition also known as lateral medullary syndrome or Wallenberg syndrome. The sympathetic nervous system generally maintains the upper eyelid position, dilates the pupils, and inhibits sweating. Any harm or disruption to these fibers can lead to the set of changes seen in Horner's syndrome. A neuromuscular junction defect, either presynaptic or postsynaptic, might result in muscle weakness causing ptosis, but that would not typically affect pupil size or sensation. Therefore, considering the symptoms and the patient's history of trauma to the neck, the most accurate explanation for this patient's symptoms would be sympathetic underactivity (F).
